# Capturing Sequences of Learners' Self-Regulatory Interactions With Instructional Material During Game-Based Learning Using Auto-Recurrence Quantification Analysis

**DOI:** 10.3389/fpsyg.2022.813677

**Published:** 2022-05-26

**Authors:** Daryn A. Dever, Mary Jean Amon, Hana Vrzáková, Megan D. Wiedbusch, Elizabeth B. Cloude, Roger Azevedo

**Affiliations:** ^1^School of Modeling, Simulation, and Training, University of Central Florida, Orlando, FL, United States; ^2^School of Computing, University of Eastern Finland, Kuopio, Finland; ^3^SoarTechnology, Inc., Orlando, FL, United States

**Keywords:** game-based learning, auto-recurrence quantification analysis, self-regulation, hierarchical modeling, eye tracking, log files

## Abstract

Undergraduate students (*N* = 82) learned about microbiology with Crystal Island, a game-based learning environment (GBLE), which required participants to interact with instructional materials (i.e., books and research articles, non-player character [NPC] dialogue, posters) spread throughout the game. Participants were randomly assigned to one of two conditions: *full agency*, where they had complete control over their actions, and *partial agency*, where they were required to complete an ordered play-through of Crystal Island. As participants learned with Crystal Island, log-file and eye-tracking time series data were collected to pinpoint instances when participants interacted with instructional materials. Hierarchical linear growth models indicated relationships between eye gaze dwell time and (1) the type of representation a learner gathered information from (i.e., large sections of text, poster, or dialogue); (2) the ability of the learner to distinguish relevant from irrelevant information; (3) learning gains; and (4) agency. Auto-recurrence quantification analysis (aRQA) revealed the degree to which repetitive sequences of interactions with instructional material were random or predictable. Through hierarchical modeling, analyses suggested that greater dwell times and learning gains were associated with more predictable sequences of interaction with instructional materials. Results from hierarchical clustering found that participants with restricted agency and more recurrent action sequences had greater learning gains. Implications are provided for how learning unfolds over learners' time in game using a non-linear dynamical systems analysis and the extent to which it can be supported within GBLEs to design advanced learning technologies to scaffold self-regulation during game play.

## 1. Introduction

Self-regulated learning (SRL) refers to learners' ability to dynamically monitor and modify their cognition, affect, metacognition, and motivation to control their learning (Winne, 2018). SRL, within this study, is captured from learners' observable events of self-regulatory processes and strategies during game-based learning. Several studies have examined how learners engage in SRL processes and employ SRL strategies to increase their learning outcomes across math (Roick and Ringeisen, 2018; Sun et al., 2018; Musso et al., 2019; Gabriel et al., 2020), reading (Snow et al., 2016; Thiede and de Bruin, 2018; Harding et al., 2019), writing (Sophie and Zhang, 2018; Nuckles et al., 2020; Sun and Wang, 2020), and science (Garcia et al., 2018; Gandomkar et al., 2020; Li et al., 2020; Taub et al., 2020) domains and technologies including hypermedia, intelligent tutoring systems, and games (Azevedo et al., 2019). In this article, we examine and analyze how learners engage in SRL behaviors as they learn within a science game-based learning environment (GBLE) to discuss how to best support learners' deployment of SRL strategies and examine the relationship between SRL behaviors and learning.

To accomplish this goal, this article: (1) defines and describes the several interacting components of SRL according to Winne's (2018) COPES model, a traditional conceptualization of SRL; (2) defines what a complex system is and defends SRL as a complex system using Winne's COPES as system components; (3) explains how SRL can be supported by GBLEs; and (4) discusses how non-linear dynamical systems theory (NDST) can measure SRL within GBLEs. From these discussions, this article introduces research questions that are grounded in and supported by the multiple theories considered in the introduction. Our ultimate goal and novel contribution to the study of SRL is the examination of dynamical SRL strategy deployment its relationship to learners' prior knowledge, agency within a GBLE, and learning outcomes, all through the lens of complex systems theory using NDST analytical tools.

## 2. Self-regulated Learning

As previously mentioned, SRL is the ability for learners to enact processes and strategies that both monitor and modulate cognitive, affective, metacognitive, and motivational processes (Winne, 2018). SRL primarily encompasses cognitive and metacognitive strategies that are deployed by the learner, such as reading instructional materials (i.e., books, research articles, posters, dialogue with non-player characters [NPCs]), gathering information important for achieving the overall goal, and retaining information required to increase domain-specific knowledge. Learners typically deploy SRL strategies throughout the phases of learning including: (1) prior to a task (i.e., forethought); (2) during a task (i.e., performance); and (3) after a task (i.e., reflection). These phases are mentioned recursively throughout SRL models and literature including Zimmerman and Moylan's (2009) SRL model, Winne and Hadwin's (2008) information-processing theory of SRL, Pintrich's (2000) model of SRL, and Nelson and Narens' (1990) metamemory framework.

To support the current article and ground the research questions, we specifically focus on Winne's (2018 conditions, operations, products, evaluations, and standards (COPES) model of SRL. This model details COPES components as occurring throughout the four phases of learning from Winne's (2018) information-processing model of SRL. This model states leaning occurs in 4 phases: (1) defining the learning task; (2) identifying and setting goals as well as plans to achieve those goals prior to interacting with their environment or starting the task; (3) deploying cognitive and metacognitive strategies that aid learners in achieving their goals; (4) adapting their learning strategies, goals, and plans to better achieve their goals. Through this COPES model, we review SRL literature that examines the relationships between learners' cognitive and task conditions, operations deployed during learning, and their products. However, this study does not incorporate evaluations nor standards when examining SRL behaviors as these were not directly measured by the learning environment. Therefore, this study specifically reviews learners' SRL behaviors in terms of how learners' conditions were related to the operations that were deployed during learning and how the interaction between these two components elicited learners' products.

### 2.1. Conditions

*Conditions* refer to the cognitive and task resources and constraints learners encounter when interacting with instructional materials. *Cognitive conditions* can include the level of prior knowledge a learner has before engaging in a learning task. Typically, learners with greater prior knowledge engage in greater SRL strategies which contribute to higher learning outcomes (Bernacki et al., 2012; Yang et al., 2018). *Task conditions* refer to constraints imposed on a learner by their environment. These constraints can refer to the environment's (e.g., game-based learning environment) restriction on learners' agency throughout the task where agency refers to learners' control over their own actions. As such, restricted agency limits the number of choices and actions a learner can perform throughout the learning process, including their deployment of SRL strategies (Bandura, 2001; Martin, 2004; Code, 2020). While full agency has been hypothesized to increase learning outcomes due to increased interest and engagement related to discovery learning (Mayer, 2004; Kirschner et al., 2006), learners are notoriously incapable of engaging in effective SRL. This is perhaps due to the difficulty of information, learners' lack of metacognitive knowledge of which SRL strategy to apply, or the open-ended nature of most learning environments (de Bruin and van Merriënboer, 2017; Schunk and Greene, 2018; Seufert, 2018; Winne, 2018; Munshi and Biswas, 2019).

### 2.2. Operations

Learners' task and cognitive conditions can influence their *operations* which refer to the cognitive strategies a learner can employ when interacting with instructional materials. The operations that are enacted center around searching for information across different sources, monitoring the learned information and their relevance toward their goal (i.e., content evaluation; Azevedo and Cromley, 2004; Greene and Azevedo, 2009; Dever et al., 2020; Azevedo and Dever, 2022), assembling several different sources into a coherent representation of information, rehearsing information in working memory, and translating information that was collected into a different type of representation (e.g., mental representation vs. concept map; Winne, 2018). Operations deployed during SRL are essential to the synthesis, (mis)understanding of information, and memorization of information for situation transfer (e.g., from virtual to classroom) and information recall. As such, it is necessary to examine how learners interact with information during SRL to examine how behaviors influence learning outcomes. Specifically, we question: How do learners' operations of selecting information throughout a complex learning task influence learning?

### 2.3. Products

*Products*, or the information that is formed using the instructional material from the environment, is perhaps the most straightforward process within the COPES framework. Simply, products can be represented by the changes in knowledge representation where products are a representation of learning. In using learning gains to represent the new knowledge learners obtain during the learning task, we can assess how the learners' task and cognitive conditions have influenced their (in)accurate deployment of operations that (dis)allowed learners to gain knowledge within a specific domain. As such, this study utilizes a formula developed by Marx and Cummings (2007; see Section 6.5) that identifies how much has been learned while accounting for learners' prior knowledge.

## 3. Defining SRL as a Complex System

SRL includes dynamically and accurately monitoring and regulating cognitive, affective, metacognitive, and motivational processes and adapting them to meet the internal (e.g., evolving understanding) and external demands and constraints of an activity (Azevedo et al., 2019). According to Favela (2020) and the assumptions of Winne's (2018) COPES model, complexity science offers a lens to understand and analyze cognitive and psychological processes that emerge as a function of complex systems. Complex systems theory describes how systems that demonstrate changing behavior due to interacting components can be explained and predicted (Favela, 2020). For the current study, we align this framework with SRL literature in which learners' conditions, operations, and products are components of SRL that change and interact with each other as learning occurs. Complex systems are generally characterized by three criteria: (1) self-organization; (2) interaction dominance; and (3) emergence (Haken, 2006; Favela, 2020).

According to these three criteria, this article argues that SRL qualifies as a complex system (see Li et al. (2022)). Constraints such as cognitive resources fluctuate with the instructional content provided in the learning environment (i.e., prior knowledge on genetic diseases vs. viruses); Operations such as cognitive strategy use shift based on task demands and goals which may change over time (McCardle and Hadwin, 2015; Cloude et al., 2021); and products are also likely to change over time as learners acquire new knowledge incrementally (Shute and Sun, 2019). While existing literature supports SRL as occurring cyclically (Winne and Azevedo, 2014; Schunk and Greene, 2018), analytical methods used within current literature does not account for the non-linear, dynamic, and complex nature of self-regulatory behaviors during learning about a difficult topic (e.g., microbiology) with a game-based learning environment. As such, it is essential to start employing complex systems theory to SRL literature to explain how learners deploy SRL strategies during learning.

*Self-organization* refers to changing behavior from which order arises out of disorder but without the influence of a central controller or programmer (Haken, 2006; Heylighen, 2008). Consistent with the concept of self-organization, SRL components mutually coordinate and constrain each other to elicit order in executed SRL behaviors which would have otherwise been chaotic (Dale et al., 2013). Initially, one may presume the central controller is the individual learner or their executive and metacognitive control functions. However, various SRL processes mutually influence one another in the context of a complex environment that may include, for example, task conditions (i.e., environmental constraints) and standards imposed on learners' processes. Moreover, learners' prior knowledge (Cognitive Conditions) can restrict which SRL strategy a learner deploys during learning. Similarly, the affordance of full agency (Task Conditions) could contribute to an unsystematic deployment of (in)accurate SRL strategies, thereby minimizing learning outcomes. In this way, processes outside of executive control interact to support SRL.

Complex systems are also characterized by their *interaction dominance* in which behavioral order and control of a system arises from the interactions between system components, not just the additive value of the components (Holden, 2009). Relative to current models of SRL, and more specifically when dealing with COPES, this characteristic of complex systems denotes the importance in considering SRL components as interactive rather than independent. Studies examining SRL have traditionally examined the impact of one component on another (e.g., Bernacki et al., 2012; Yang et al., 2018), but rarely have SRL studies examined the dynamic relationship between components. Under the interaction dominance characteristic of complex systems, there is not just an additive or unidirectional relationship between system components which elicit a certain behaviors. Rather, SRL is possible through the interaction between cognitive and metacognitive strategies across time and SRL phases. It is important to note that since SRL is theoretically aligned with complex systems, there is much to be gained from leveraging analytical techniques based in complex systems theory (i.e., NDST) that can extract the very nature of dynamically interacting components.

Similarly, although definitions vary, the criteria of *emergence* often refers to how the behavior of an entire system cannot be broken down into just the sum of the components (Favela, 2020). In other words, the behavior of the whole system supersedes the behaviors of the individual components. In the case of COPES, this means that SRL cannot be isolated into either conditions, operations, or products. Additionally, SRL cannot be broken into separate cognitive and metacognitive strategies as SRL requires the oscillation of all components and both types of strategies throughout the learning process. The conceptualization of SRL as a complex system is made increasingly evident when we consider non-traditional environments with high levels of learner-environment interactivity such as that found during game-based learning.

## 4. Supporting SRL During Game-Based Learning

The goal of a game-based learning environment (GBLE) is to make multimedia instructional materials accessible in a non-linear fashion which increases agency during learning *via* the deployment of SRL strategies while maintaining the interest, engagement, and motivation of a learner (Clark et al., 2016; Sawyer et al., 2017; Mayer, 2019; Plass et al., 2019; Shute and Sun, 2019; Taub et al., 2020). Because of this, GBLEs are increasingly being used in order to support learning through their combination of (1) narrative to increase engagement and interest, (2) tasks to support domain learning, and (3) game elements to promote engagement with both the task and the instructional materials presented throughout the environment. This uniquely positions learners within GBLES, relative to other learning environments, to have the agency to control their learning progression and direction without having too much freedom they are overwhelmed by choice.

During game-based learning, it is essential for learners to engage in SRL strategies to meet the demands of learning activities and comprehend instructional materials essential for attaining domain knowledge in pursuit of a goal (Winne and Azevedo, 2014). Although, the open-ended nature of GBLEs both facilitates and limits the successful use of SRL strategies. On one hand, GBLEs allow learners agency to engage in and develop self-regulation through goal-setting and the use of monitoring and cognitive strategies (e.g., reading, note-taking, summarizing) and tools (e.g., instructional materials, help-seeking; Winne and Hadwin, 2013; Nietfeld, 2018). Alternatively, the open-ended nature may not provide the needed support for the learner to coordinate the several cognitive and metacognitive strategies required for successful SRL (Josephsen, 2017). Because of this, there is a need for GBLEs to be developed with scaffolds that guide learners' interactions with instructional materials to simultaneously support successful SRL and increase domain-specific learning gains. The balance between support and freedom provided by GBLEs calls for the incorporation of a complex systems theory concept, far-from-equilibrium.

### 4.1. Far-From-Equilibrium Systems

Adapting the concept of far-from-equilibrium from physical sciences, behavior can be described as learners' patterns of, or oscillations between, stable and unstable states (Veerman et al., 2021). That is, healthy cognitive systems, such as learners' SRL behaviors, are demonstrated by behaviors which maintain a balance between stability (i.e., rigidity) and adaptability (i.e., chaotic). To support this healthy behavior, the GBLE should promote the balance of SRL behaviors that are not too rigid (i.e., no agency) nor too chaotic (i.e., discovery-based learning). A too-rigid SRL system would demonstrate a greater repetition of SRL strategies during learning, such as only attending to one instructional material (i.e., book, research article, non-player character), perhaps promoted through the restricted agency imposed by the GBLE. Behaviors which could be too chaotic would demonstrate significantly greater novelty not conducive to content learning, potentially encouraged through full agency afforded to learners by the GBLE.

Applying the far-from-equilibrium concept of complex systems theory, healthy SRL behaviors should be demonstrated by learners' balance between stable and adaptable SRL strategies and actions during learning with a GBLE. This balance can be supported and maintained through cognitive conditions available to (i.e., prior knowledge) and task conditions imposed on (i.e., restricted agency) the learner. Task resources and constraints include the environmental features and mechanics that directly influence how a learner will interact with instructional materials within the GBLE. To guide learners' interactions with instructional materials, a GBLE may intentionally restrict the amount of agency learners have while still promoting their freedom in choosing the SRL strategies to be deployed. While agency as scaffolds (i.e., restricted agency as guiding learners throughout the GBLE) have been found to increase learning outcomes (Sawyer et al., 2017; Dever and Azevedo, 2019a; Dever et al., 2020), we must ask if agency promotes a healthy balance between rigidity and adaptability as learners deploy SRL strategies to interact with instructional materials in a GBLE. A methodological approach to study this question is to use a non-linear dynamical systems theory (NDST) analytical method for understanding learners' SRL behavioral shifts during learning with a GBLE.

### 4.2. A Non-linear Dynamical Systems Approach to Measuring SRL

NDST describes how numerous interacting components have a multiplicative effect on system-level behavior, where small changes in component processes can produce sudden (non-linear) behavioral shifts (Riley and Holden, 2012; Amon et al., 2019). Because of this, NDST can be used to evaluate and measure the repetition and predictability in learners' SRL strategy use, denoting the degree to which a learners' SRL strategy use throughout a GBLE follows the far-from-equilibrium concept. Due to the interdependent nature of non-linear dynamical systems, global behavior both constrains and is constrained by its underlying component processes, such that reciprocal feedback entrains processes at various levels (Amon et al., 2019). Because SRL behaviors change over time due to the constantly changing interactions with GBLEs as well as the acquisition of domain-specific knowledge, SRL can be measured using an NDST approach. While NDST has yet to be used to understand SRL with GBLEs from a complex systems theory stance, a study by Garner and Russel (2016) has applied NDST and sequence-oriented techniques to understand how learners deploy SRL while reading multiple texts. This study found differences of recurrent patterns between learners who took notes vs. those who did not while reading instructional materials. Building on the findings from this study, this article acknowledges the complex SRL strategies that occur during game-based learning and based on a GBLE's environmental affordances of agency.

This study utilizes auto-recurrence quantification analysis (aRQA), an NDST method, to examine how learners adaptively shift between repetitive and novel sequences of interactions with a GBLE. This method is also used to describe the relationship between these sequences and task conditions, learning gains, and SRL ability. aRQA quantifies the degree of repetition or “recurrence” within a single time series (Webber and Zbilut, 2005), indicating the extent to which a system returns to the same states across various time lags. Because NDST is a central part to studying the relationship between agency and SRL behaviors within this study, it is important to understand how learners' time series data is used to identify SRL behavioral patterns.

**Figure 1:**
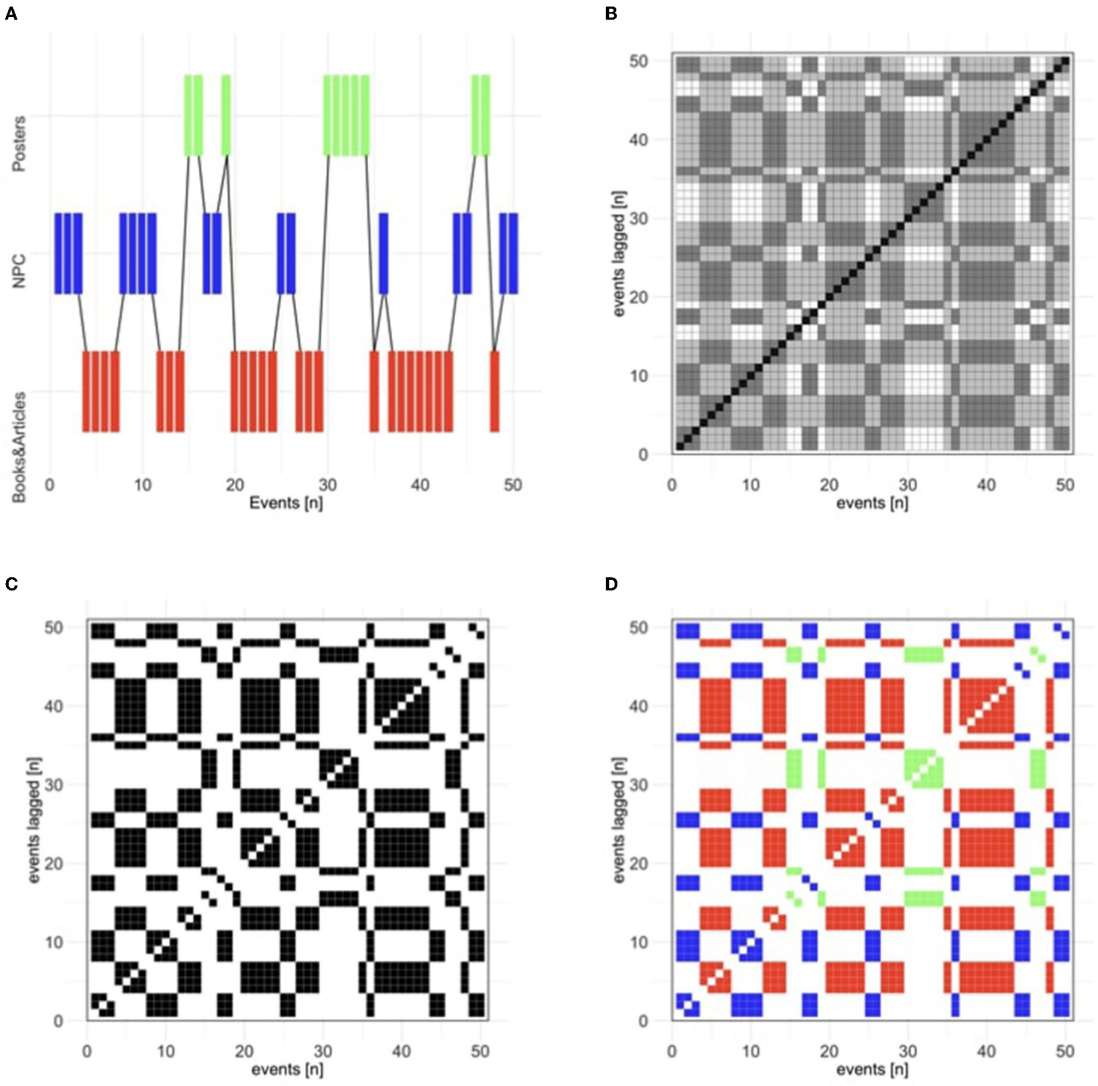
**(A)** Time series of events in the chronological order (or events on the main diagonal) that are transformed into the **(B)** distance matrix, **(C)** recurrence plot, and finally, into the **(D)** color-coded recurrence plot (Books in red, NPC in blue, and Posters in green).

Figure 1A demonstrates the time series of the events in chronological order. Figure 1B shows how RQA first transforms a participant's time series—in this case, with categorical data—into the Figure 1B distance matrix representing the Euclidean distance between the values that represent areas of interest where participants were looking (i.e., Books, Posters, or NPC). When a participant is looking at the same area of interest at two different time points (e.g., time points t1 and t8), then the recurrent state is highlighted black. The diagonal represents the line of identity (LOI), where the time series is recurrent with itself at lag 0. Diagonal lines parallel to the LOI represent successively greater time lags between the points that are being compared in terms of distance. The Panel C recurrence matrix is created by applying a radius parameter that defines the threshold at which points are considered sufficiently similar enough to be considered recurrent. Thus, a very small radius value is used such that only exact matches are counted as recurrent, such that the recurrence matrix highlights points where the same area of interest is returned to at different time lags. Unique to the authors' approach (e.g., Amon et al., 2019; Necaise et al., 2021), we include an additional procedure to “color-code” the matrix (Figure 1D) to identify the distinct behaviors that underlie the recurrent points in the matrix.

We examine an RQA metric called percent determinism (DET), where determinism refers to the relative predictability of the system; i.e., the extent to which the system's future state can be predicted by the system's current state. In terms of RQA, DET technically refers to the percentage of the points that form diagonal lines, representing repeated sequences of behavior. For example, a time series with areas of interest *A, B, C, A, B, A* would include one recurrent sequence (A, B) depicted as a two-point diagonal on either side of the LOI. For this study, we use learners' interactions with instructional materials (i.e., books, research articles, posters, non-player characters) which hold all information needed to develop domain knowledge. Specifically, learners' interactions with these instructional materials are represented by learners' operations or time-evolving strategies that they deploy during gameplay and dynamically alter to fit their present needs. For the purposes of our study, more repetitive behavioral sequences of instructional material may give insight into how deployed SRL strategies interact with cognitive and task conditions to result in learners' products, or learning outcomes. Thus, aRQA provides a unique lens through which to understand SRL in terms of how task and cognitive conditions are related to how learners interact with instructional materials and the resulting learning gains.

## 5. Current Study

While previous studies have examined SRL using NDST methods (Garner and Russell, 2016), few studies in SRL literature: (1) examine how SRL strategies are deployed during game-based learning (Cloude et al., 2020; Taub et al., 2020; Dever et al., 2021); (2) operationalize SRL as a sequence of dynamic, temporally unfolding processes and examine the direct relationships between these processes simultaneously using eye tracking data; and (3) use an NDST approach to analyzing how SRL occurs during learners' time in a GBLE. The goal of this study was to address these gaps in current literature by examining SRL using the lens of complex systems theory and analytically investigate how learners use SRL strategies within a GBLE through applying NDST methods. To address these gaps and further the SRL field conceptually, methodologically, and analytically, we propose three research questions:

**Research Question 1: To what extent do learners' SRL behaviors and dwell times differ across instructional material throughout gameplay?** This first research question examines how long a learner dwelled, or attended to, instructional materials, and how this duration varied as a function of relative game time, type of instructional material, and relevance of the instructional material to the pre-test. As prior studies have shown that learners are typically unable to engage in meaningful SRL and accurately deploy SRL strategies that will significantly increase their learning gains (Josephsen, 2017), we hypothesize that there will be significant main and interaction effects to explain within-person variability, but do not assume a direction. However, as individual differences (e.g., prior knowledge, task conditions, etc.) can significantly change how learners deploy SRL strategies during game-based learning, we propose that there will be significant between-person variability in the relevant vs. irrelevant instructional material dwell times.

**Research Question 2: To what extent are learners' task and cognitive conditions, learning outcomes, and sequences of SRL behaviors with instructional material related to dwell times on instructional materials throughout gameplay?** This second research question builds off of the first research question and aims to understand the full picture of how SRL processes can be examined and related to each other when examining eye gaze dwell times across relevant and irrelevant instructional materials. First, we hypothesize that learners with restricted agency will have greater learning gains than those with full agency, as supported by previous literature (Bradbury et al., 2017; Sawyer et al., 2017; Dever and Azevedo, 2019a; Dever et al., 2020). Further, we hypothesize that learners with restricted agency, greater prior knowledge, and greater learning gains will demonstrate increased dwell times on relevant instructional materials as they can better evaluate content relevance. It is possible that a relationship between the experimental manipulation and subsequent learning gains is a product of constrained interaction and, in turn, more repetitive eye gaze sequences. As such, we further hypothesize that learners with more repetitive sequences of SRL behaviors with instructional materials will have greater gaze dwell times on relevant, rather than irrelevant, instructional materials.

**Research Question 3: How do learners' task conditions, cognitive conditions, and learning gains relate to their sequences of SRL behaviors?** This research question is used to explore how learners differ in how often learners deploy repetitive sequences of SRL behaviors between task and cognitive conditions and its relationship with learning gains. For this research question, we hypothesize that learners with more repetitive eye-gaze sequences (i.e., more rigid behaviors), will be associated with restricted agency but related with higher learning gains. Further, we hypothesize that learners with higher prior knowledge will demonstrate more novel behaviors as they use instructional material interaction diversity as an SRL strategy to keep far-from-equilibrium interactions.

## 6. Methods

### 6.1. Participants and Materials

A total of 139 undergraduate students were recruited from a large public university based in the United States to learn with a narrative-centered, game-based learning environment called Crystal Island (Rowe et al., 2011; Dever et al., 2020, 2021; Taub et al., 2020). Crystal Island was designed to foster (1) higher-order thinking skills, such as effective problem solving and scientific reasoning, while also gaining knowledge about (2) microbiology content. For purposes of this article, a subsample of 82 undergraduates (68.3% female; *M*_age_ = 20.1, *SD*_age_ = 1.69) were included in the analysis based on meeting the following criteria: (1) completed the entire study with Crystal Island; (2) were randomly assigned to either the full or partial agency conditions; (3) had no prior experience interacting with Crystal Island before participating in the study; and, (4) did not have missing data points across all converging data channels captured before, during and after game-based learning, including both log files and performance measures (e.g., pre/post-test assessments).

Most participants reported their race as “White/Caucasian”(68.30%; *n* = 56), while the remaining reported “American Indian or Alaskan Native” (1.22%; *n* = 1), “Asian” (12.20%; *n* = 10), “Black or African American” (7.32%; *n* = 6); “Hispanic or Latino” (7.32%; *n* = 6), and “Other” (3.66%, *n* = 3). The subsample also indicated that they “Did not play video games at all” (18.29%; *n* = 15), “Rarely played video games” (35.37%; *n* =29), “Occasionally played video games” (21.95%, *n* = 18), “Frequently played video games” (15.85%; *n* = 13), and “Very frequently played video games” (58.54%; *n* = 7). The subsample also reported having “No video game skills” (14.63%; *n* = 12), “Limited skills” (21.95%; *n* = 18), “Average” (37.80%; *n* = 31), “Skilled” (20.73%; n = 17), and “Very skilled” (4.88%; *n* = 4). The majority of the sample indicated they played a total of “0–2” (68.29%; *n* = 56), “3–5” (13.41%; *n* = 11), “5–10” (7.32%; *n* = 6), “10–20” (9.76%; *n* = 8), and “Over 20” (1.21%; *n* = 1) hours per week. This study was approved by the university's Institutional Review Board before recruiting participants and informed consent was gathered before collecting data.

**Figure 2:**
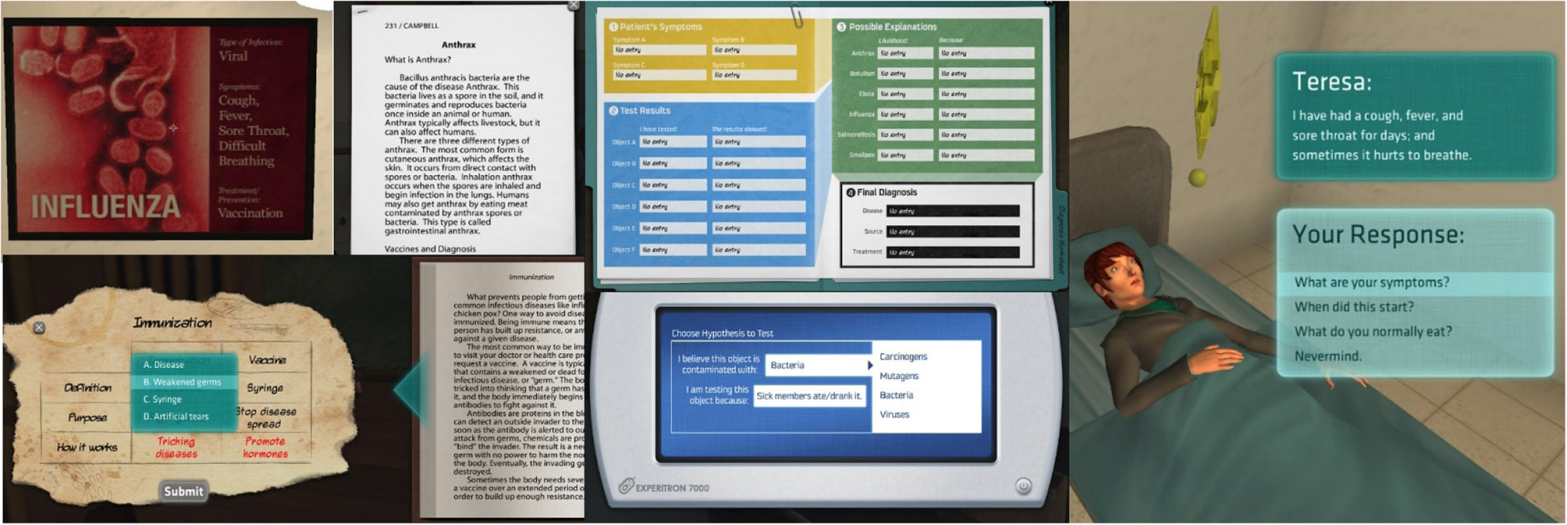
Elements within the Crystal Island Environment.

To assess participants' understanding of microbiology, a 21-item, 4-option multiple choice, pre/post-test assessment was administered before and after game-based learning with Crystal Island see Figure 2, regardless of whether or not participants successfully solved the mystery. The assessments were designed with 12 factual (e.g., “*What is the smallest type of living organism?*”) and 9 procedural items (e.g., “*What is the difference between bacterial and viral reproduction?*”). Participants answered between 6 and 18 correct items across on the pre-test assessment (*Med* = 11, *M* = 55%, *SD* = 0.14), while participants answered between 9 and 19 correct items (*Med* = 14, *M* = 67%, *SD* = 0.12) on the post-test assessment (Rowe et al., 2011). In addition to the knowledge assessments, several self-report items were administered before and after the learning session but these data were not analyzed in this article. Game play duration ranged from 39.73 to 135 min (*M* = 85, *SD* = 19).

### 6.2. Experimental Design

In this study, participants were randomly assigned to one of two experimental conditions: (1) full agency (*n* = 47), and (2) partial agency (*n* = 35). These groups were built to experimentally manipulate the learners' level of control (i.e., agency) in the sequence of interactions with game features built into Crystal Island. In the control condition–i.e., full agency, participants were given complete control over their sequence of interactions with Crystal Island, or business-as-usual. Participants in the experimental condition–i.e., partial agency, were given restricted control over their sequence of interactions (e.g., first reading a book and then generating a hypothesis), meaning they were required to initiate a specific order of actions to progress with the learning session. For example, participants in the partial agency condition were required to first visit Kim, an NPC nurse in the camp infirmary. Once they entered the infirmary, the participant could not leave until all items within the building were interacted with (e.g., clicked on with no minimum time requirement). Once able to leave the infirmary, the next building was “unlocked.” This experimental condition was designed around a particular sequence of interactions that scaffolded higher-order thinking skills such as effective problem solving and scientific reasoning activities *via* game features and restricted agency.

It is important to note that between the conditions, dwell times on instructional materials (i.e., how long participants looked at instructional materials indicated by eye-gaze behavior) were not restricted other than the requirement that learners in the partial agency interact with the material in some way (i.e., they could select the book, but not attend to it according to eye-tracking metrics). Additionally, all types of instructional materials were found in each building, so participants in the partial agency condition were not restricted to certain types of instructional materials as they progressed in the game.

Across all participants, participants spent an average of 86.0 min (*SD* = 19.5 minutes) in game where learners in the full agency condition spent an average of 80.2 min (*SD* = 20.1 min) and those in the partial agency conditions spent an average of 93 min (*SD* = 15.7 min).

### 6.3. Procedure

Participants were recruited using flyers across a large North American public university campus. Once participants were scheduled, they were instructed to come into the university laboratory space to obtain informed consent and complete the experiment for up to 2 h. A CITI-certified researcher greeted the participant upon their arrival and instructed them to sit at the experimental station which consisted of a computer, keyboard, and mouse. After informed consent was obtained, they were randomly assigned to one of two conditions. Participants were then instructed to complete a series of questionnaires including the pre-test assessment to gauge their level of microbiology science content understanding and self-report items on emotions, motivation, and presence.

Afterwards, the researcher calibrated participants to three apparati: (1) SMI EYERED 250 eye tracker using a 9-point calibration to capture their eye movements during game-based learning (SMI, 2014), (2) facial recognition software to measure their facial expressions of emotions (), as well as (3) electrodermal activity bracelet called Empatica 4 to capture their physiological arousal and stress response (iMotions, 2015). Specifically, the participant was required to view a gray screen with a neutral expression for approximately 10 s to establish a baseline for the facial recognition software and EDA bracelet. Once successful calibration was completed, participants started learning and problem solving with Crystal Island. Participants were given up to 90 min to solve the mystery. Once they completed the game, or they engaged with Crystal Island for 90 min, participants were instructed to stop what they were doing and complete a similar set of post-test items and self-report measures including the post-test assessment on microbiology. Upon their completion, participants were debriefed about the objectives of the study and their participation, thanked, and paid $10/h for their time.

### 6.4. Apparatus

Eye gaze behaviors were recorded using a table-mounted SMI EYERED250 eye tracker (sampling rate = 250 Hz). Participants were calibrated with a 9-point calibration. Participants' fixation durations, saccades, and regressions on different areas of interests (AOIs), which define the boundaries on the computer screen where specific elements or information are held. To be classified as a fixation duration, the participant was required to have relatively stable gaze behavior for at least 250 ms. These data were captured continuously using iMotions software 2015 as participants engaged in game-based learning.

### 6.5. Coding and Scoring

*Reading dwell times and instances* were established using gaze behaviors and log files. Log files collected as learners engaged with Crystal Island identified the times at which instructional materials were opened denoted by log file timestamps using event-based recording. As from just log files alone researchers cannot assume that learners were reading information from the instructional material, eye gaze behavior was used to supplement the identification of reading instances. Learners' total fixation durations on a single AOI while the instructional material was opened denoted by log files were aggregated into dwell times which identifies the total time learners spent fixating on a single AOI instance. These AOIs were laid overtop of each type of instructional material including books and research articles, posters, and the dialogue boxes as well as the NPC itself to identify NPC instances.

*Learning gains* were operationalized using normalized change scores (Marx and Cummings, 2007) which identified participants' differences in pre- and post-test scores proportional to the number of total points possible and controlling for participants' prior knowledge, or pre-test score.

*Content evaluations* were operationalized by first identifying the relationship between instructional materials and pre-test questions. Instructional materials which directly addressed a question on the pre-test were identified as relevant. If the information did not address a pre-test question, the instructional material was identified as irrelevant as the information within the text was not needed to increase learning gains. While content evaluations were not directly observable, we take the stance that learners who attend to relevant materials are making a correct content evaluation whereas attending to irrelevant instructional materials were incorrect content evaluations. This classification of relevant vs. irrelevant instructional materials is based on prior SRL (Azevedo et al., 2004) and priming literature (McNamara, 2005) where it is assumed that participants exposed to microbiology information on the pre-test may identify the same information within the GBLE as more important, and therefore more relevant to their learning. Across a total of 40 instructional materials spread throughout the Crystal Island environment, 19 were classified as relevant. Specifically, 33% (3/9) of NPCs, 57% (12/21) of books and research articles, and 40% (4/10) of posters were considered relevant to the pretest [see (Dever et al., 2021)].

*Relative game time* was calculated by taking the time at which an interaction occurred and dividing that by a participant's total time in game so that all interactions were scaled as occurring from Time = 0 to Time = 1. For example, if a participant opened a book at Time = 300s, and they spent 2382s in game, then the participant opened that book 12.6% into their game. This allows for a uniform comparison across all participants in terms of their total time spent interacting with the game.

### 6.6. Statistical Processing

To process the data and conduct analyses, several packages in R (R Core Team, 2017), including its base package, were used. For the multilevel modeling and basic reporting of statistics we used the “lme4” (Bates et al., 2015), “jtools” (Long, 2018), and “emmeans” (Lenth et al., 2018) packages. To conduct aRQA analyses and obtain the output, we utilized the “crqa” (Coco et al., 2020) package in R.

### 6.7. Model Building and Estimation

To examine how participants' sequences of SRL behaviors in reading and evaluating instructional material during game-based learning differs within and between learners, we constructed a multilevel growth model including several observation- and individual-level variables. Specifically, our overall model examined how dwell times on instructional materials (i.e., outcome variable) is influenced by observation- and individual-level variables. The dependent variable of dwell time was log transformed (with a base of 10) to normalize the data and reduce heteroscedasticity (skew and kurtosis < |2|). Due to the log transformation, reported estimates of the independent variables are geometric means where the estimates are exponentiated.

After transformation, several leveraging outliers (*N* = 72 out of 4,346 total observations) were removed from analyses as the dwell times of these instances fell outside a 1.5 interquartile range of the first and third quartiles of data. After the transformations and outlier removal, two-level multilevel linear growth models were used to analyze the hierarchically structured data where observations (*N* = 4,274) were nested within individual learners (*N* = 82). Throughout their time in game, each learner had an average of 52.12 observations (*SD* = 9.98with the number of observations ranging from 25 to 74 across all learners. Prior to exploration of observation- and individual-level variables, an unconditional means (null) model was estimated. This model demonstrated an intraclass correlation coefficient (ICC) of 0.05, suggesting that 5% of variation in instructional material dwell times is between learners [*t*_(82.6)_ = 2.99, *p* < 0.01]. This justifies our use of multilevel linear growth models to examine the observation- and individual-level variables influencing dwell times on instructional materials.

#### 6.7.1. Observation-Level Variables

These variables included relative game time, the type of instructional material, and the relevance of the material to the pre-test. Because participants varied in the total amount of time they interacted with the game, relative game time scales each participants' time in game from 0 to 1 where the raw game time a participant initiated an action was divided by the participants' total time in game. The values of relative game time were then forced to zero for each participant to interpret the model intercepts. In other words, participants' first initiation of an action was treated as a zero (with all other interactions adjusted accordingly) so that the growth model intercept, originally representing the dwell time where time was equal to zero which does not have a meaningful value, now represents the dwell time of participants' first time interacting with an instructional material. The type of instructional material included books and research articles (informative text, no visuals), non-player characters (informative text, uninformative visuals), and posters (informative and uninformative text and visuals) that provided information about microbiology concepts.

All types of instructional material were evaluated for their relevance in relation to microbiology concepts introduced in the pre-test. For example, an item on the pre-test asks “*How do vaccines protect you?*”. For this question, a book or research article on the function of vaccines would be relevant to the pre-test whereas an instructional material on genetic diseases is irrelevant for this question. The classification of an instructional material is based on priming literature (McNamara, 2005) where participants are assumed to classify (either accurately or inaccurately) instructional material as either relevant or irrelevant in reference to the pre-test (Dever et al., 2020, 2021).

#### 6.7.2. Individual-Level Variables

These variables include participants' condition, their prior knowledge, and the percent determinism of their sequences of instructional material interactions. Within the models, both variables were treated as fixed. Condition refers to either the full or partial agency conditions that participants were randomly assigned prior to interacting with the Crystal Island environment (see Section 6.2). Prior knowledge in microbiology was calculated using participants' raw pre-test scores on their microbiology content quiz before interacting with instructional materials in the Crystal Island environment. Percent determinism represents the proportion of recurrent sequences within a single time series, denoting the predictability of a system where a greater proportion of recurrent sequences indicates a system with higher behavioral predictability.

An unconditional means model was run to examine the variation of the dependent variable between individuals. The model found a 0.05% intraclass correlation coefficient; in other words, 5% of variation in the dwell times on instructional materials in Crystal Island is between learners [*t*_(82.6)_ = 2.99, *p* < 0.01] and 95% is within learners. As such, several other multilevel models were constructed including: (1) an unconditional growth model with the latent time variable as an independent variable; (2) observation-level variables and their interactions; (3) significant predictors from (2) and individual-level variables; and (4) predictors from (3) and cross-level interactions.

## 7. Results

### 7.1. Research Question 1: To What Extent Do Learners' SRL Behaviors and Dwell Times Differ Across Instructional Material Throughout Gameplay?

For Research Question 1, we examined the unconditional growth model (i.e., Model 1) and the growth model with observational-level predictors (i.e., Model 2). Model 1 examined how time influenced the dwell time across all instructional materials. From this model, the dwell time on participants' initial interaction with instructional material was approximately 31.5s (*SD* = 52.7) which was significantly different from zero [*t*_(211.4)_ = 66.6, *p* < 0.01]. However, dwell time across all instructional materials decreased by 68.0% (S.E. = 0.08) as participants' time in game progressed [*t*_(4236.1)_ = −14.4, *p* < 0.01] from participants' initial interaction with instructional material. Model 1 fits the data significantly better than the unconditional means model [*BIC* = 14387.9, *D* = 14,354; X^2^_(1)_ = 202.1, *p* < 0.01] where, by adding a latent time variable, the growth model explains approximately 4% of individual-level variance in dwell time.

Model 2 (*BIC* = 12,623, *D* = 12,506) incorporated observational-level variables (i.e., type of instructional material, relevance of the instructional material to the pretest) in addition to the latent time variable to examine the effect on the variation in participants' dwell times. This model was a statistically significant better fit than the unconditional growth model [X^2^_(10)_ = 1,848, *p* < 0.01]. Holding all other variables constant, learners' average fixation durations on instructional materials was 104.6s (SE = 0.08). There were significant main and interaction effects for and between all variables. For every unit increase in relative game time, dwell times decreased by approximately 89.0% [S.E. = 0.16; *t*_(653.86)_ = −13.36, *p* < 0.01].

Overall, participants had significantly greater dwell times on relevant (*M* = 48.4 s; *SD* = 56.5 s), rather than irrelevant (*M* = 37.1 s; *SD* = 48.8 s), instructional materials [*t*_(4186.7)_ = 3.37, *p* < 0.01] by approximately 25.9% (S.E. = 0.07). Books and research articles (*M* = 77.3 s; *SD* = 64.3 s) had greater dwell times than dialogue with NPCs by 85.6% [S.E. = 0.07; *M* = 22.7; *SD* = 17.5; *t*_(4200.0)_ = −26.8, *p* < 0.01] and posters by 91.5% [S.E. = 0.09; *M* = 8.96; *SD* = 5.33; *t*_(4210.6)_ = −28.3, *p* < 0.01]. In relation to dwell times on instructional materials during participants' time in game, dwell times on books and research articles decreased by 88.9% (S.E. = 0.16) as time in game increased. Compared to books and research articles, dwell times on posters and dialogues on NPCs increased at a greater rate as the game progressed by 6-fold [S.E. = 0.20; *t*_(4209.54)_ = 8.86, *p* < 0.01] and 9-fold [S.E. = 0.19; *t*_(4211.7)_ = 11.85, *p* < 0.01], respectively.

When examining the relationship between participants' content evaluations, type of instructional material, and game time on dwell times, Model 2 found that participants' dwell time on pretest-relevant instructional materials decreased by 56% (S.E. = 0.18) as participants learned with Crystal Island [*t*_(4182.4)_ = −4.65, *p* < 0.01]. When examining a three-way interaction and controlling for observation-level variables, dwell times on relevant posters [S.E. = 0.16; *t*_(4180.0)_ = 2.65, *p* < 0.05] and dialogues with NPCs [S.E. = 0.17; *t*_(4176.8)_ = 6.70, *p* < 0.01] increased as participants engaged with Crystal Island by 98.5 and 97.1% respectively compared to dwell times on books and research articles.

### 7.2. Research Question 2: To What Extent Are Learners' Task and Cognitive Conditions, Learning Outcomes, and Sequences of SRL Behaviors Related to Dwell Times on Instructional Materials Throughout Gameplay?

#### 7.2.1. Task and Cognitive Conditions

An independent samples t-test was first run to ensure that prior knowledge did not differ between experimental conditions. Results were not significant (*p* > 0.05), so we included both as individual-level variables. However, when running Model 3 which contained the observation-level variables from Model 2 and added prior knowledge and agency conditions as individual-level variables, there was not a main effect for either condition or prior knowledge (*p* > 0.05). When examining cross-level effects of prior knowledge and condition, only the interaction between condition and type of instructional material was significant where participants in the partial agency condition had significantly greater dwell times on posters than participants in the full agency condition by approximately 29% [S.E. = 0.10; *t*_(160.1)_ = 2.57, *p* < 0.01]. No other interaction effects were significant. Therefore, we conclude that task and cognitive conditions do not significantly relate to the dwell time on both relevant and irrelevant instructional materials as the game progresses.

#### 7.2.2. Learning Outcomes

Model 4 added normalized learning gain as an individual-level variable to Model 3. However, the model did not find a significant main effect or interaction effect when adding learning gains to the model. As such, we conclude the learning outcomes are not significantly related to the dwell time on either relevant or irrelevant instructional materials as the game progresses.

#### 7.2.3. Sequences of SRL Behaviors

For Model 5, percent determinism was added as an individual-level variable to Model 3. Percent determinism has a significant main effect where, with all other variables constant, for every unit increase in percent determinism, dwell times decreased by approximately 2.0% [S.E. = 0.01; *t*_(113.0)_ = −2.68, *p* < 0.05]. There was one cross-level interaction between percent determinism and type of instructional material where, compared to dwell times on books and research articles, for every unit increase of percent determinism, dwell times on posters increased by approximately 2.0% [S.E. = 0.01; *t*_(4097.2)_ = 2.62, *p* < 0.05], with no significant relationship between NPC dialogue and percent determinism (*p* > 0.05). From these results, we conclude that there is a significant relationship between percent determinism and the dwell times spent on instructional materials regardless of participants' content evaluations.

### 7.3. Research Question 3: How Do Learners' Task Conditions, Cognitive Conditions, and Learning Gains Relate to Their Sequences of SRL Behaviors?

Information on the recurrent sequences of books and research article opens, NPC dialogues, and poster interactions were extracted from the lags outputted from aRQA analyses (see Figure 1 for example). This information was used to first calculate the total number of recurrent points across all participants and instructional material types (see Table 1).

**Table 1:** Proportional means of recurrence points across Lags 1-5 and instructional materials.

**Recurrent action**	**Lag1**	**Lag2**	**Lag3**	**Lag4**	**Lag5**
NPCs	0.212	0.231	0.262	0.275	0.264
Books and research articles	0.468	0.482	0.529	0.548	0.617
Posters	0.320	0.287	0.210	0.177	0.119

To examine how the dynamics (i.e., sequences) of instructional material interactions, cognitive conditions, and task conditions influence learning, frequencies of learners' recurrent points across Lags 1-3 were first correlated against each other to ensure multicollinearity does not affect the outcome of further comparisons. Several significant correlations existed between Lags 1–3 and across the instructional materials (*p* < 0.01), so Lag1 frequency counts of recurrent points across all instructional materials were used as variables for hierarchical clustering. Using this method, three clusters of participants were identified differing in the number of recurrent sequences of instructional materials on Lag1. Cluster 3 was removed from subsequent analyses as there were less than 10 participants (*N* = 5), the remaining clusters, Cluster 1 (*N* = 34) and Cluster 2 (*N* = 43), were used in further analyses. T-tests revealed that learners classified within Cluster 1 had significantly fewer book and research article recurrent points as well as poster recurrent points compared to learners classified within Cluster 2, but no significant difference in NPC dialog interaction recurrent points (see Table 2).

**Table 2:** Recurrent point frequency between clusters 1 and 2.

**Instructional material**	**Cluster 1**	**Cluster 2**	***t*****-value;** ***p*****-value**
	**[M(SD)]**	**[M(SD)]**	
NPCs	1.35 (1.07)	1.33 (0.75)	*t*_(56.7)_ = 0.13; *p* > 0.05
Books and research articles	2.21 (0.77)	3.88 (1.10)	*t*_(74.1)_ = −7.88; *p* < 0.01
Posters	1.56 (0.82)	2.65 (0.98)	*t*_(74.3)_ = −5.40; *p* < 0.01

Using both Clusters 1 and 2 as a predictor, a multiple linear regression was run to understand how the cluster learners were classified within as well as their agency within Crystal Island influenced learning gains. Prior knowledge was not included as (1) prior knowledge does not differ between conditions; and (2) prior knowledge did not significantly interact with any variables in the hierarchical linear model (see RQ2). Overall, there was a significant multiple linear regression model [*F*_(3, 37)_ = 4.79; *p* < 0.01] that accounted for 16% of variance. The multiple linear regression found a significant main effect of cluster where, keeping condition constant, participants classified as Cluster 2 (*M* = 0.45; *SD* = 0.24), with greater recurrent points on both books and research articles and posters, had significantly greater learning gains than those in Cluster 1 (*M* = 0.33; *SD* = 0.28) with less recurrent points (*t* = 2.58; *p* < 0.05). There was a second main effect of condition where, keeping cluster constant, participants in the partial agency condition (*M* = 0.48; *SD* = 0.25) had significantly greater learning gains than learners with full control over their own actions (*M* = 0.33; *SD* = 0.26; *t* = 3.11; *p* < 0.01). A significant interaction effect was also observed (*t* = −2.05; *p* < 0.05).

From this interaction, participants classified within Cluster 1 and with full agency had a significantly greater learning gains than participants in Cluster 2 with full agency. Specifically, participants within Cluster 1 with full agency had a mean learning gain of 0.23 (*SD* = 0.26) whereas participants in Cluster 2 with full agency had a mean learning gain of 0.43 (*SD* = 0.23). Meanwhile another significant effect was found where participants within the partial agency condition had a mean learning gain of 0.52 (*SD* = 0.23) if they were classified within Cluster 1, but a mean learning gain of 0.47 (*SD* = 0.26) if they were classified within Cluster 2.

In summary, results across all research questions have several main findings: (1) dwell times on instructional materials as a function of learners' content evaluations of instructional materials over gameplay where dwell time on pre-test relevant materials decrease; (2) the predictability of SRL behaviors, denoted by percent determinism, is related to learners' greater dwell times on instructional materials; and (3) learner profiles of recurrent instructional material sequences can be extracted and are related to both agency and overall learning outcomes where learning gains are greatest in participants who had restricted agency and greater recurrent interactions with instructional materials.

## 8. Discussion

As very few studies have provided a comprehensive analysis of unfolding SRL processes during game-based learning, the goal of this study was to examine the emergence of SRL from a complexity science perspective. This article investigated whether cognitive strategies, task conditions, and SRL behaviors, grounded within Winne's (2018) COPES model of SRL, moderated when and for how long learners gathered information during learning with a GBLE. This study viewed SRL through the lens of complex systems theory and analyzed SRL using an NDST technique to understand how SRL should be scaffolded within GBLEs through restricted agency.

The first research question examined how dwell times on both irrelevant and relevant instructional materials vary as a function of relative game time, type of instructional material, and relevance of the instructional material. Overall, hypotheses for the first research questions were supported where significant between- and within-person variability were identified. Further, dwell times across all instructional materials decreased over learners' time in game and there were generally greater dwell times on relevant than irrelevant instructional materials. This could potentially be due to the familiarity with materials over the course of gameplay, indicating more accurate metacognitive monitoring SRL behaviors. Even though dwell times on books and research articles were significantly greater than both NPC dialogues and posters, the dwell times on NPCs and posters increased at a greater rate compared to books and research articles as learners interacted with Crystal Island.

Of most interest is the interaction between relative game time and instructional material relevance. Specifically, dwell times on pre-test relevant materials generally decreased over learners' gameplay whereas dwell times on relevant NPCs and posters increased over learners' time in game. From these results, we conclude that while learners are initially able to accurately deploy SRL strategies for information-gathering by engaging with pre-test relevant instructional materials, as time engaging in game-based learning progressed, learners' ability to consult relevant information from irrelevant books and research articles decreased. Because dwell times on books and research articles did not change during learning but time on relevant books and research articles decreased, we infer that the long blocks of text without any supporting diagrams or conversational interactions did not support learners' deployment of accurate SRL monitoring strategies (i.e., content evaluations). However, learners were generally able to deploy accurate content evaluations when interacting with posters and NPCs as they learned with Crystal Island. Our results expand prior studies such as that by Dever et al. (Dever and Azevedo, 2019b) and Taub et al. (2018) by including relative game time, dwell times, and content evaluations based on relevance to domain knowledge acquisition. These results support SRL as a complex system through and add to Winne's (2018) IPT of SRL model by examining how operations can affect how learners interact with their learning environment and how this can be captured and measured using eye-tracking and log-file data.

The second research question expanded previous results to understand how SRL processes can be examined and related to each other when examining eye gaze dwell times across relevant and irrelevant instructional materials. Hypotheses were partially confirmed where results did not find that task conditions, cognitive conditions, or learning outcomes were significantly related to dwell times on either relevant or irrelevant instructional materials during learning with Crystal Island. However, hypotheses regarding SRL sequencing behaviors were partially confirmed where the models found that as percent determinism increases, the dwell times on instructional materials increase regardless of material relevance to the pre-test. This effect may have implications for the oscillation between accurate and inaccurate use of SRL strategies due to the non-significance in dwell times on relevant and irrelevant instructional materials. Further, this result is interesting as learners who repeat sequences of information-gathering behaviors with instructional materials tend to have greater dwell times on these materials. To fully explore this effect, future analyses should examine the differences in repeated behaviors for each type of instructional material.

From these results, we conclude that SRL systems with greater predictability and less novel behaviors typically have greater dwell times across instructional materials. The findings contradict research conducted on task conditions, cognitive conditions, and overall learning which found these constructs to significantly interact. This is potentially due to how SRL within this study was measured using an NDST method to examine the stability vs. rigidity of SRL behaviors individually rather than aggregating using typical parametric methods. However, these results contribute to the dynamic and complex conceptualization of SRL as we were able to identify a positive relationship between the predictability of SRL behaviors and learning outcomes. Specifically, this result has implications for (1) Winne's (2018) model to include learners' recursive interactions with GBLE elements as an operational strategy for SRL, and (2) scaffolding design through the lens of far-from-equilibrium concept within complex systems theory. For example, treating SRL systems as complex should extend to theory as well as how GBLEs are designed. From the results of the study, GBLEs should increase the minimum time of instructional material interaction and promote learners' use of several different types of representations while still structuring their approach to how learners interact with the environment. Scaffolds within GBLEs should be designed to balance learners' exploratory behaviors with the structure provided by scaffolds to encourage behaviors that follow the far-from-equilibrium concept.

To further explore learners' sequences of instructional material interactions and how they relate to task conditions, cognitive conditions, and learning gains, the third research question extracted information from the aRQA output. In doing so, we were able to explore how learners differ in (1) the distribution of novel behavioral sequence indices over different instructional materials; and (2) the novelty of behavioral sequences between task and cognitive conditions and its relationship with learning gains. For this third research question, we hypothesized that learners with more repetitive eye gaze sequences would be present in learners with restricted agency and related with higher learning gains. Further, we hypothesized that learners with higher prior knowledge would demonstrate more novel behaviors as they used instructional material interaction diversity as an SRL strategy. Specifically, more novel behaviors denote a healthier SRL system, and as such, the use of multiple different types of materials can be considered a learning strategy employed by learners.

Overall, our hypotheses were not confirmed as prior knowledge was not included within our analyses due to previous non-significant relationships. However, when clustering all participants according to the frequency of recurrent points on Lag 1 and between all instructional materials, hypotheses were confirmed. First, we were able to identify differences between learners where two clusters identified learners as having greater books and research article recurrence (Cluster 2) or fewer recurrence in these interactions (Cluster 1) with no differences in the frequency of NPC recurrent points. From our analyses, learners who had restricted control over their own actions (i.e., the partial agency condition) demonstrated significantly greater learning gains, regardless of classified cluster profiles than learners with full control. However, when ranking the significant clusters and conditions in reference to overall learning, we conclude that learners with partial agency in Cluster 1 had greater learning gains, **demonstrating novel behavior while engaging in guided game-based learning increases overall learning gains**. These results are parallel to findings for the concept of agency (Sawyer et al., 2017; Dever et al., 2020; Taub et al., 2020) but are novel by examining learners' recursive behaviors in gathering information during game-based learning. These results are consistent with the far-from-equilibrium concept of complex systems theory which promotes the balance between rigidity (i.e., partial agency) and chaos (i.e., novel SRL behavior).

## 9. Future Directions and Concluding Statement

Our findings have significant conceptual, theoretical, methodological, empirical, and design implications for future research on SRL and GBLEs. Conceptually, our use of NDST analytical methods to analyze SRL process data during game-based learning significantly contributes to the field of SRL and learning technologies by including complex systems theory (Lajoie et al., 2018; Jarvela and Bannert, 2019). While much has been published describing SRL as a dynamic, temporally unfolding process, there is no published research using complex systems theory as a theoretical grounding or dynamical systems in modeling as a method to examine the dynamics of SRL strategies, specifically information-gathering behaviors, during GBLEs (Azevedo et al., 2019; Plass et al., 2019; Favela, 2020). That is, SRL has theoretically been described as temporally dynamic, with some models assuming non-linearity as well, but we extend these assumptions by positing SRL as a complex system and used NDST analytics to empirically support this claim. To date, this article acts as one of the first studies to apply NDST methods to SRL using complex systems theory (see Garner and Russell, 2016; Li et al., 2022).

The use of non-linear dynamical systems techniques allows researchers to specify, operationally define, and make predictions about assumptions regarding the dynamics of SRL processes. More specifically, we can understand how the dynamics of each SRL process (within and across different data channels) are connected to specific complex SRL components described in Winne s COPES model. A dynamical systems approach ties each of the COPES together elegantly and produces testable hypotheses that need to be further explored by researchers (e.g., how do other cognitive conditions such as motivation or emotions relate to how learners oscillate between more recursive or novel operations?)

In addition, our findings using log-files and eye movements provide evidence of the dynamics of specific cognitive and metacognitive processes that, until recently, could only be described in an abstract manner using models such as Winne's (2018) theory of SRL. More specifically, our findings indicating that relationships between eye gaze dwell time and (1) the type of representation a learner gathers information from (i.e., large sections of text, poster, or dialogue); (2) the ability of the learner to distinguish relevant from irrelevant information; (3) learning gains; and (4) agency, could only have been established using the non-linear dynamical systems modeling and statistical techniques used in our study. As such, our findings, based on our use of multimodal data, can begin to augment current models of SRL (e.g. Winne, 2018) by adding the micro-level processes (e.g., judgments of learning, monitoring progress toward goals) that are currently hypothesized to predict learning and performance. Dynamical system modeling can be used to study task and cognitive conditions and affordances of the GBLEs (e.g., agency) as learners engage in SRL processes.

Future research should focus on how other multimodal data (e.g., physiological and facial expressions of emotions) contribute to our understanding of the dynamics of other key SRL processes such as affect and motivation. Can the dynamics capture subtle states or state transitions related to emotion regulation, emotion regulation efficacy, etc. (McRae and Gross, 2020)? What are the multimodal data that most accurately predict affective and motivational states? What specific indices can be extracted from each data channel to understand the temporal dynamics of affect and motivation during GBLE? Would non-linear dynamical modeling techniques and analytical approaches predict that the same states within and across data channels are predictive of learning, reasoning, performance, etc.? How would learning technology-specific affordances impact the dynamics of SRL across learning technologies? For example, how would the lack of autonomy embodied into an intelligent tutoring system impact the dynamics of cognitive, affective, metacognitive, and motivational SRL processes compared to a simulation?

Researchers should consider longer and different types of experiments to test how changing agency, number and types of relevant and irrelevant instructional materials, behavioral repertoire of the NPCs, etc. would impact learners' self-regulation and multimodal data. This new research strategy would also force researchers to isolate the exact dependent variables for each data channel and how they both individually and collectively contribute to our understanding of the dynamics of SRL across learners and contexts.

Our findings also have implications for the design of future GBLEs where NPCs can detect when, how, and why learners fluctuate in their accurate SRL strategy deployment. Further, complex systems theory lends support in the development of GBLEs to support the balance between rigid and complex SRL behaviors. The system's intelligence capability could lead the NPCs to engage in a conversation with the learners about why their ability to identify relevant text has changed. Further, this could serve as an opportune time to pedagogically intervene by providing different types of scaffolding or prompting to the learners. We see several innovative pedagogical interventions delivered by the NPCs. For example, “Your eye movements suggest that you are not spending enough time on the relevant textual cues. Would you like for me to model these processes? Or, would you like for me to show you your multimodal data to show you what, where, and how you have changed your overall strategy?”. In summary, the use of non-linear dynamical system modeling has tremendous potential to advance the field of SRL, multimodal data, and GBLEs.

## Data Availability Statement

The datasets presented in this article are not readily available because of written consent restraints. Requests to access the datasets should be directed to Daryn Dever, corresponding author.

## Ethics Statement

The studies involving human participants were reviewed and approved by North Carolina State University International Review Board. The participants provided their written informed consent to participate in this study.

## Author Contributions

DD significantly contributed to the conceptualization and construction of this manuscript as well as the analyses that were conducted. MA and HV contributed their expertise of complex systems and non-linear dynamical systems theory and the application of auto-Recurrence Quantification Analysis. MW contributed to the analyses and data extraction as well as to the editing of this manuscript. EC wrote the methodology section and provided edits. RA significantly contributed his expertise in SRL and supported the future directions and final conclusion section. All authors contributed to the editing of the manuscript.

## Funding

This study was supported by funding from the National Science Foundation (DUE#1761178 and DRL#1661202) and the Social Sciences and Humanities Research Council of Canada (SSHRC 895-2011-1006).

## Author Disclaimer

Any opinions, findings, conclusions, or recommendations expressed in this material are those of the author(s) and do not necessarily reflect the views of the National Science Foundation or the Social Sciences and Humanities Research Council of Canada.

## Conflict of Interest

EC was employed by SoarTechnology, Inc. The remaining authors declare that the research was conducted in the absence of any commercial or financial relationships that could be construed as a potential conflict of interest.

## Publisher's Note

All claims expressed in this article are solely those of the authors and do not necessarily represent those of their affiliated organizations, or those of the publisher, the editors and the reviewers. Any product that may be evaluated in this article, or claim that may be made by its manufacturer, is not guaranteed or endorsed by the publisher.
